# Paper-Based DNA Biosensor for Rapid and Selective Detection of miR-21

**DOI:** 10.3390/bios14100485

**Published:** 2024-10-08

**Authors:** Alexander Hunt, Sri Ramulu Torati, Gymama Slaughter

**Affiliations:** 1 Center for Bioelectronics, Old Dominion University, Norfolk, VA 23508, USA; 2 Department of Electrical and Computer Engineering, Old Dominion University, Norfolk, VA 23508, USA

**Keywords:** gold inkjet printing, microRNA-21 detection, paper-based biosensor, gold nanoparticles

## Abstract

Cancer is the second leading cause of death globally, with 9.7 million fatalities in 2022. While routine screenings are vital for early detection, healthcare disparities persist, highlighting the need for equitable solutions. Recent advancements in cancer biomarker identification, particularly microRNAs (miRs), have improved early detection. MiR-21 is notably overexpressed in various cancers and can be a valuable diagnostic tool. Traditional detection methods, though accurate, are costly and complex, limiting their use in resource-limited settings. Paper-based electrochemical biosensors offer a promising alternative, providing cost-effective, sensitive, and rapid diagnostics suitable for point-of-care use. This study introduces an innovative electrochemical paper-based biosensor that leverages gold inkjet printing for the quantitative detection of miR-21. The biosensor, aimed at developing cost-effective point-of-care devices for low-resource settings, uses thiolated self-assembled monolayers to immobilize single-stranded DNA-21 (ssDNA-21) on electrodeposited gold nanoparticles (AuNPs) on the printed gold surface, facilitating specific miR-21 capture. The hybridization of ssDNA-21 with miR-21 increases the anionic barrier density, impeding electron transfer from the redox probe and resulting in a current suppression that correlates with miR-21 concentration. The biosensor exhibited a linear detection range from 1 fM to 1 nM miR-21 with a sensitivity of 7.69 fM µA^−1^ cm^2^ and a rapid response time (15 min). With a low detection limit of 0.35 fM miR-21 in serum, the biosensor also demonstrates excellent selectivity against interferent species. This study introduces an electrochemical paper-based biosensor that uses gold inkjet printing to precisely detect miR-21, a key biomarker overexpressed in various cancers. This innovative device highlights the potential for cost-effective, accessible cancer diagnostics in underserved areas.

## 1. Introduction

Cancer remains the second leading cause of death globally, with approximately 9.7 million fatalities in 2022 [1]. Public health initiatives promoting routine screenings have been crucial in early detection efforts. Despite these initiatives, disparities in healthcare access and screening technologies persist, necessitating equitable healthcare access to address this global challenge [2]. The recent advancements in cancer biomarker identification have significantly improved early detection and treatment outcomes [3]. Among the various cancer biomarkers, microRNAs (miRs) have shown great potential due to their presence in easily accessible body fluids (blood, urine, saliva, etc.) [4,5,6]. MiRs are small, non-coding RNAs that regulate gene expression, influencing key cellular processes like proliferation and apoptosis [7]. Their dysregulation in cancer cells classifies them as either oncogenic miRs (onco-miRs) or tumor-suppressive miRs (TS-miRs), making them vital for cancer diagnostics [8]. Therefore, identifying and quantifying miRs offer invaluable insights into cancer diagnostics, enabling the early detection of malignancies and providing prognostic indicators for disease progression [9,10].

MiR-21, an onco-miR, is highly expressed in various cancers, including breast, lung, and prostate. It inhibits apoptosis and thereby promotes cancer cell survival [11,12]. This makes miR-21 a valuable pan-cancer diagnostic tool, offering clinicians a versatile means of identifying and stratifying patients based on their cancer status [13,14]. Traditional miR detection methods, such as northern blotting, microarrays, and quantitative real-time polymerase chain reaction (qRT-PCR), are accurate but have limitations like time-consuming protocols, high sample requirements, and the need for expensive equipment and skilled technicians [15,16,17,18,19], consequently limiting their utility, particularly in resource-limited settings [19]. Electrochemical biosensors have emerged as a promising alternative, offering high sensitivity, selectivity, simplicity, and rapid analysis capabilities, suitable for point-of-care (POC) applications [20,21,22,23]. However, the economic limitations of large-scale fabrication of traditional biosensing platforms highlight the need for cost-effective manufacturing methods [24,25].

Paper-based biosensors offer economic and environmental advantages, including low cost and ease of fabrication, making them suitable for POC diagnostic platforms [26,27,28]. Using paper substrates reduces the reliance on expensive materials and sophisticated fabrication instrumentation, making them highly suitable for decentralized and affordable POC diagnostic platforms [29]. Unlike paper-based colorimetric biosensors (i.e., dipsticks, lateral flow assays, etc.), paper-based electrochemical biosensors do not require costly labeling techniques for quantifying analytes. Moreover, inkjet printing and screen printing have been shown to enhance scalability and utility [30,31,32] while incorporating gold nanoparticles (AuNPs) into electrode designs. These approaches significantly enhance the electrochemical biosensor performance by providing a high surface area-to-volume ratio, excellent biocompatibility, and superior catalytic activity [33,34].

Herein, we introduce a novel, paper-based electrochemical biosensor for quantitatively detecting miR-21, utilizing gold inkjet printing on photopaper (PhP). AuNPs electrodeposition process was utilized to effectively address any insulating gaps from the sintering of the gold ink, thereby enhancing current output and surface area for bioreceptor immobilization. By immobilizing complementary ssDNA-21 onto electrodeposited AuNPs on gold-printed photopaper electrodes (PhP-Au/AuNPs), the fabricated biosensor achieved selective detection of miR-21 with a linear range from 1 fM to 1 nM with a sensitivity of 7.69 fM µA^−1^ cm^2^ and an impressively low detection limit of 0.35 fM in serum. These findings underscore the potential for mass production of POC devices for cancer diagnostics using PhP-Au/AuNPs as the electrode material. This method is not only cost-effective and straightforward but also ideal for large-scale production, making it highly suitable for POC cancer diagnostics in resource-limited settings. This biosensor represents a significant advancement in accessible and reliable diagnostics, capable of transforming healthcare delivery by facilitating early disease detection and monitoring.

## 2. Materials and Methods

### 2.1. Chemicals and Solutions

Sulfuric acid (H_2_SO_4_), sodium chloride (NaCl), and fetal bovine serum were purchased from Thermo Fisher Scientific, Waltham, MA, USA. Potassium ferricyanide (K_3_[Fe(CN)_6_]), Gold (III) chloride trihydrate (HAuCl_4_·3H_2_O), potassium chloride (KCl), potassium phosphate monobasic (KH_2_PO_4_), sodium phosphate dibasic (Na_2_HPO_4_), tris(2-carboxyethyl)phosphine (TCEP), and the gold foil were purchased from Sigma-Aldrich, USA. All oligonucleotide sequences (Table 1) were purchased from Integrated DNA Technologies (IDT), Coralville, IA, USA. The JG-106 gold ink was purchased from Novacentrix, Austin, TX, USA. The polyimide (PI) tape was purchased from MYJOR, Shanghai, China. All solutions were prepared using deionized water (18.2 MΩ-cm). 0.01 M phosphate buffered saline solution pH 7.4 (1× PBS) was prepared with 137 mM NaCl, 2.7 mM KCl, 1.8 mM KH_2_PO_4_, and 10 mM Na_2_HPO_4_. All oligonucleotide and TCEP solutions were prepared in 1× PBS. 

### 2.2. Apparatus and Instrumentation

The Dimatix Materials Printer DMP-2850 (Fujifilm, Inc., Tokyo, Japan) was employed to print gold electrodes on photopaper. A conventional three-electrode setup was used, with the gold photopaper electrode serving as the working electrode, Ag/AgCl as the reference electrode, and a platinum wire as the counter electrode. Electrodeposition of AuNPs was carried out using direct current potential amperometry (DCPA) with the EC Epsilon EClipse™ Potentiostat (Bioanalytical Systems, Inc., Louisville, KY, USA). Cyclic voltammetry (CV) was performed with square wave voltammetry (SWV) at a scan rate of 100 mV s^−1^ and a potential range from −0.1 to 0.4 V (potential step of 4 mV, amplitude of 15 mV, and frequency of 1 Hz) to quantitatively assess the miR-21 hybridization event within the same potential range. All electrochemical tests were performed in triplicate. Scanning electron microscopy (SEM) was conducted using the JSM-IT700HR (JEOL Ltd., Peabody, MA, USA). 

### 2.3. Fabrication of PhP-Au/AuNPs Electrode

**Figure 1:**
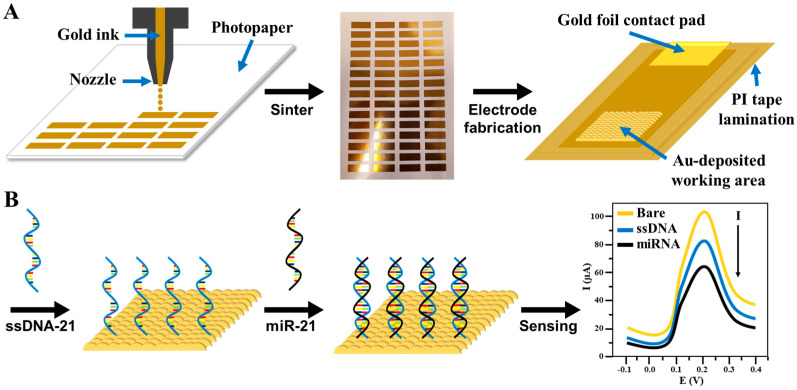
Schematic illustration of (**A**) electrochemical PhP-Au/AuNPs biosensor fabrication process and (**B**) ssDNA-21 immobilization and hybridization with target miR-21 sensing process. Electrode fabrication is further detailed in the Supplemental Information.

Four layers of gold ink were printed onto the photopaper to fabricate the 60-electrode array (Figure 1A). Following printing, the array was air-dried overnight at room temperature in a fume hood. Subsequently, it underwent sintering at 140 °C for 30 min in a conventional oven. Inkjet printing parameters for electrode fabrication are provided in the Supplementary Information. Individual gold inkjet-printed photopaper electrodes (PhP-Au) were then prepared by cutting them out. Each PhP-Au electrode had a 3 × 3 mm piece of gold foil affixed as a contact pad, with the working area defined using PI tape, as illustrated in Figure S1 of the Supplementary Materials. The PhP-Au electrode underwent electrochemical cleaning with 0.05 M sulfuric acid via cyclic voltammetry for 10 cycles, employing a potential range from −0.1 to 1.5 V and a scan rate of 50 mV s^−1^. After cleaning, the electrode was thoroughly rinsed with DI water to remove any residual acid and impurities from the electrode’s surface and was subsequently stored in a desiccator to dry. AuNPs were then electrodeposited onto the working surface of the PhP-Au electrode (Figure 1A). This involved submerging the PhP-Au working electrode, along with external Ag/AgCl reference and platinum wire counter electrodes, in a 2 mM HAuCl_4_ solution. Electrodeposition of AuNPs onto the PhP-Au working area was achieved via DCPA at an applied voltage of −0.5 V for one hour, resulting in the PhP-Au/AuNPs electrode. Following deposition, the PhP-Au/AuNPs electrode was thoroughly rinsed with deionized water and stored in a desiccator for future use.

### 2.4. ssDNA-21 Immobilization and miR-21 Hybridization

To selectively detect miR-21, the complementary aptamer ssDNA-21 probe was immobilized onto the PhP-Au/AuNPs electrode surface using a thiol linker (Figure 1B). Initially, a solution of TCEP and ssDNA-21 was prepared at a concentration ratio of 100X TCEP to ssDNA-21 concentration. The solution was vigorously vortexed for one minute at 15-min intervals, totaling four times, at room temperature, and subsequently stored at 4 °C for future use. TCEP, an effective reducing agent, cleaves disulfide bonds to generate free thiol groups. These thiol groups form robust bonds with gold surfaces via gold-thiol chemistry, ensuring the secure attachment of the ssDNA-21 to the PhP-Au/AuNPs electrode. A 5 µL aliquot of ssDNA-21/TCEP solution was drop-cast onto the working area of the PhP-Au/AuNPs electrode and placed in a sealed humidity chamber. The reaction proceeded for 3 h at room temperature [35]. Post-incubation, the surface of the ssDNA-21-modified PhP-Au/AuNPs electrode (PhP-Au/AuNPs/ssDNA) was thoroughly rinsed with 1× PBS. To prevent drying and denaturation of the ssDNA-21 bioreceptor, a droplet of 1× PBS was added to the working area, and the electrode was stored in the humidity chamber at 4 °C. The PhP-Au/AuNPs/ssDNA electrode was gently rinsed with 1× PBS, and the excess PBS on the working area surface was carefully aspirated, ensuring no contact with the working surface. Subsequently, 5 µL of miR-21 solution at varying concentrations (ranging linearly from 1 fM to 1 nM) was drop-casted onto the center of the PhP-Au/AuNPs/ssDNA working area and incubated for 15 min at room temperature in a humidity chamber. Following incubation, the electrode’s working surface was thoroughly rinsed with 1× PBS, and SWV was performed is the presence of varing miR-21 concentrations. Figure 1B provides a schematic overview of the ssDNA immobilization and the miR-21 sensing process.

## 3. Results and Discussion

### 3.1. Characterization of PhP-Au/AuNPs Electrode Surface Morphology

**Figure 2:**
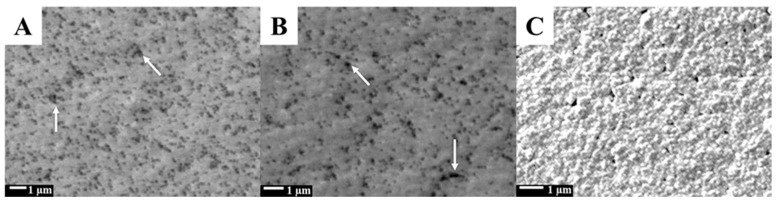
SEM microgrphs of (**A**) PhP-Au before sintering, (**B**) after sintering for 30 min at 140 °C, and (**C**) after electrodeposition. White arrows highlight insulating gaps.

SEM imaging was conducted to analyze the morphological characteristics of the PhP-Au electrode at various stages: pre-sintering (Figure 2A), post-sintering (Figure 2B), and after AuNP electrodeposition (Figure 2C). Gold inks typically comprise AuNPs stabilized with surface ligands or capping agents that maintain particle separation and prevent aggregation during printing [36]. Initially, when the gold ink was printed onto photopaper, these particles remained discrete due to ink formulation and printing conditions [37]. Therefore, the printed gold ink particles remained separated as there was insufficient energy to bring them into close contact, creating insulating gaps that prevent a continuous conductive path [38]. Sintering applies thermal energy to desorb or decompose the surface ligands, enabling particle fusion and a continuous conductive network formation (Figure 2B). This transformation enhances electrical conductivity noticeably. Post-sintering, the PhP-Au electrode demonstrated improved electrical continuity compared to its pre-sintered state. Before sintering, the gold ink electrode exhibited high resistance (1.5 MΩ), which impeded the electrical current flow. Upon sintering, the electrode resistance was reduced to 3 Ω, allowing for enhanced current conduction that improves the biosensor’s signal quality, sensitivity, and response time. While sintering effectively converted discrete gold ink particles into a conductive network, it further introduces insulating gaps or pores [39] that can compromise electrode surface uniformity and create competitive functionalization sites, potentially interfering with ssDNA-21 immobilization. Pretreatment with sulfuric acid aided in removing residual contaminants, oxidation layers, and other impurities from these gaps post-sintering. Additionally, electrodeposition of AuNPs served to fill these insulating gaps, resulting in a more homogeneous and conductive surface as depicted in Figure 2C. This AuNPs-layer also enhanced the electrode’s effective surface area, providing more active sites for electron transfer reactions, improving biosensor sensitivity and efficiency.

Moreover, the increased surface area facilitated the immobilization of a larger quantity of ssDNA-21, which is crucial for enhancing miR-21 detection. Thus, AuNPs electrodeposition established a robust platform for specific capture and detection of miR-21, ensuring high performance of the paper-based electrochemical biosensor.

### 3.2. Electrochemical Characterization

**Figure 3:**
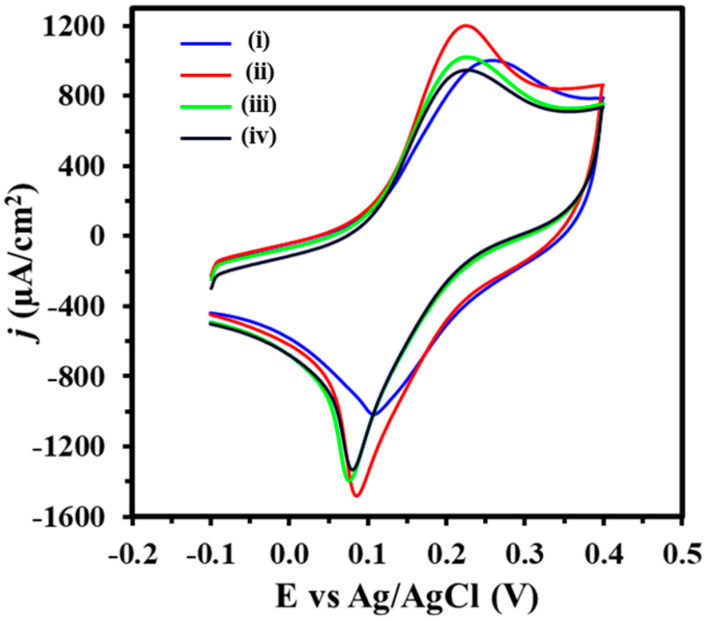
Cyclic voltammogram (CV) of PhP-Au (i), PhP-Au/AuNPs (ii), PhP-Au/AuNPs/ss-DNA (iii), PhP-Au/AuNPs/ss-DNA/miR-21 (iv) electrode. Voltammograms were obtained in 5 mM (K_3_Fe(CN)_6_^4−/3−^) + 0.1 M KCl with a scan rate of 100 mV s^−1^.

Figure 3 illustrates that the PhP-Au electrode exhibited a peak oxidation current density of 1004.5 µA cm^−2^ (Curve i). Following the electrodeposition of AuNPs onto the PhP-Au, there was a notable enhancement in electrode performance, with the peak oxidation current density increasing to 1202.5 µA cm^−2^ (Curve ii). This enhancement was attributed to the increased surface area provided by the small-sized AuNPs, which improved electrode conductivity and promoted more efficient electron transfer kinetics between the electrode surface and the redox reporter.

The immobilization of 7.5 µM ssDNA-21 played a crucial role in establishing a robust and sensitive biosensing interface to ensure the proper orientation and availability of the DNA for target miR-21 binding, thereby enhancing detection selectivity and sensitivity. Upon immobilization on the AuNPs-modified electrode, the phosphate backbone of nucleic acids introduced negative charges to the electrode’s surface. These negative charges created an electrostatic anionic barrier that hinders electron flow from the potassium ferricyanide redox probe [40], which is evident as a decrease in peak oxidation current density from 1202.5 to 1004.5 µA cm^−2^ (Curve iii). This confirmed successful probe immobilization and served as a metric for monitoring hybridization events. Upon hybridization of 10 pM miR-21 with the complementary ssDNA-21, the resulting formation of double-stranded complexes increased the density of negative charges within the anionic barrier, further impeding electron flow (Curve iv), thus confirming successful miR-21 hybridization to the ssDNA-21.

**Figure 4:**
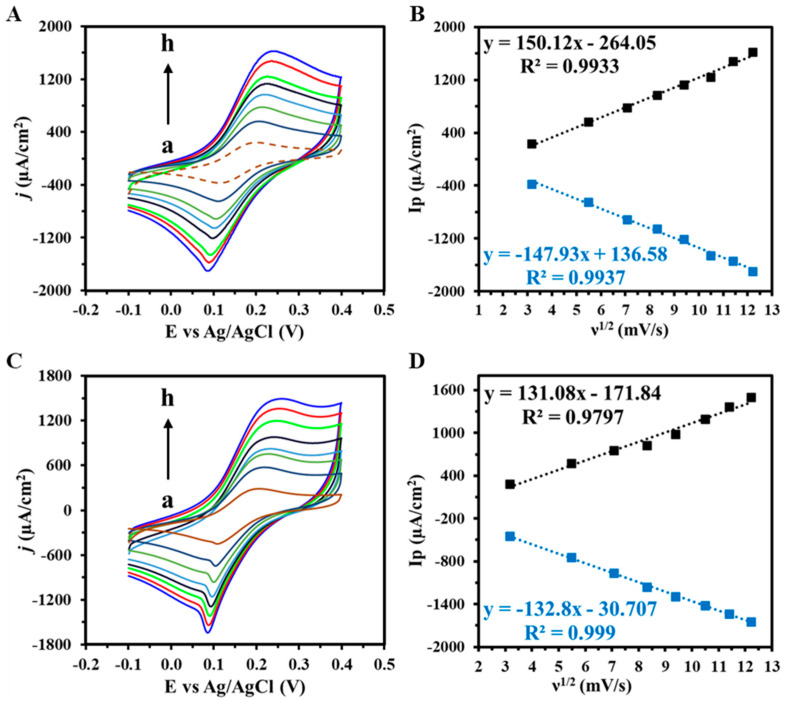
CV response of (**A**) PhP-Au/AuNPs electrode and (**C**) PhP-Au/AuNPs/ssDNA electrode from varying scan rates (a–h: 10–150 mV s^−1^) with (**B**,**D**) corresponding linearity plot of peak anodic and cathodic current densities vs. square root of scan rates (ν). Voltammograms were obtained in 5 mM (K_3_Fe(CN)_6_^4−/3−^) + 0.1 M KCl. A dashed line indicates the baseline current.

To assess whether the reaction follows a diffusion-controlled or surface (adsorption) controlled process, a variation in CV scan rates from 10 to 150 mV s^−1^ was conducted on both PhP-Au/AuNPs and PhP-Au/AuNPs/ssDNA electrodes (Figure 4). The relationship between peak current density (Ip) and scan rate is pivotal in electrochemical analysis. In both cases, PhP-Au/AuNPs (Figure 4A) and PhP-Au/AuNPs/ssDNA (Figure 4C), the Ip showed a linear dependence on the square root of the scan rate (ν) (Figure 4B,D), which is indicative of a diffusion-controlled process [41]. To further confirm diffusion control, log(peak oxidation current density (Ia)) was plotted against log(scan rate) for both the PhP-Au/AuNPs (Figure S2A) and the PhP-Au/AuNPs/ssDNA electrodes (Figure S2B), yielding slopes of 0.6953 and 0.5972, respectively. This further reinforced the diffusion-controlled nature of the electrochemical processes [20].

Interestingly, the reduction current density curve exhibited a narrower shape with slightly greater peak current density than the broader oxidation curve observed for PhP-Au/AuNPs and PhP-Au/AuNPs/ssDNA electrodes, suggesting an underlying electrochemical process. The distinct peak shapes and current density differences implied varying kinetics between the reduction and oxidation processes. The narrower, higher current peak during reduction suggested a faster and more efficient electron transfer, possibly indicating a more kinetically favorable process for the reduction reaction than oxidation. Conversely, the broader oxidation peak with lower current density may indicate greater influence from diffusion limitations. This disparity could stem from differences in diffusion coefficients of oxidized and reduced species or concentration gradients near the electrode surface during the redox cycle. Additionally, the asymmetry in peak shapes and currents might be influenced by surface adsorption phenomena or residual impurities from the gold ink. It is expected that strong adsorption of reduced species onto the electrode surface could enhance current density and sharpen peaks due to rapid electron transfer, while easier desorption of oxidized species could lead to broader, lower current oxidation peaks due to slower electron transfer and mass transport effects.

### 3.3. Optimization of ssDNA-21 Concentration and miR-21 Hybridization Time

**Figure 5:**
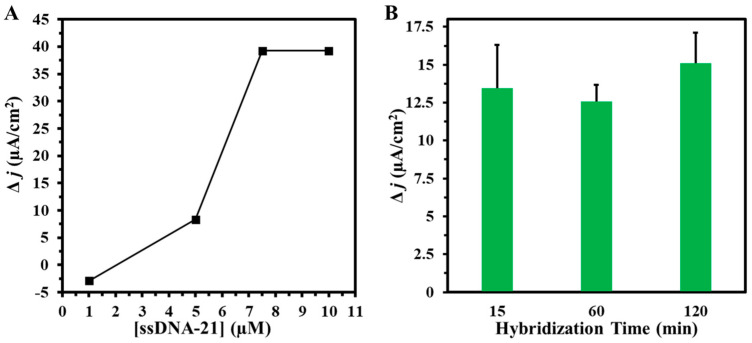
(**A**) Change in current density (Δ*j*) vs. various ssDNA-21 concentrations and (**B**) Δ*j* vs. various miR-21 hybridization times. Voltammograms were obtained in 5 mM (K_3_Fe(CN)_6_^4−/3−^) + 0.1 M KCl. Hybridization experiments were conducted in duplicates.

The concentration of ssDNA-21 immobilized on the PhP-Au/AuNPs electrode surface was optimized to maximize sensitivity while minimizing non-specific binding events of the paper-based electrochemical biosensor. Four independent PhP-Au/AuNPs electrodes were each drop-casted with 5 µL of ssDNA-21 solutions at concentrations of 1 µM, 5 µM, 7.5 µM, and 10 µM onto the center of the working area, followed by a 3-h incubation at room temperature. Post-incubation, each electrode underwent thorough washing with 1× PBS and was used as the working electrode for subsequent SWV measurements. Figure 5A illustrates the difference in SWV peak current density before and after immobilization of varying ssDNA-21 concentrations. The immobilization of ssDNA-21 at a low concentration of 1 µM did not induce a significant change in current density output, indicating inadequate attachment of the bioreceptor to the working surface. However, at 5 µM ssDNA-21, there was an observable increase of 8 µA cm^−2^ in current density, suggesting successful bioreceptor attachment. To ascertain the saturation point of ssDNA on the electrode surface, concentrations of 7.5 µM and 10.0 µM were evaluated. A maximum current density of 39 µA cm^−2^ was achieved at 7.5 µM, with no further increase observed upon increasing to 10.0 µM, indicating saturation of the electrode surface with ssDNA at 7.5 µM. Therefore, subsequent variations in miR-21 concentration were assessed using electrodes immobilized with 7.5 µM ssDNA-21 to ensure the reliability and accuracy of our biosensor.

Following the optimization of ssDNA-21 concentration, the hybridization time for miR-21 was optimized to achieve rapid yet precise detection. Three independent PhP-Au/AuNPs electrodes were functionalized with 5 µL of 7.5 µM ssDNA-21 drop-casted onto the center of the working area and incubated for 3 h at room temperature in a humidity chamber. Subsequently, 5 µL of 1 fM miR-21 was applied to the working electrode surface and incubated in the humidity chamber for 120, 60, or 15 min. Each hybridization trial was conducted in duplicate. Following hybridization, the PhP-Au/AuNPs/ssDNA electrode was washed with 1× PBS and used as the working electrode for SWV measurements. Figure 5B illustrates the difference in SWV peak current density before and after varying miR-21 hybridization times. Allowing a two-hour hybridization time resulted in a significant change in Δ*j* (current density) of 15 µA cm^−2^, highlighting the biosensor’s capability to detect miR-21 by transducing the hybridization event into an electrochemical signal. However, such a prolonged experimental duration of 120 min is impractical for POC diagnostics where rapid response times are crucial. Consequently, the 60 min hybridization time resulted in a Δ*j* of 12.5 µA cm^−2^. Further reducing the hybridization time to 15 min, a more feasible option for POC devices, yielded a substantial change in Δ*j* of 13.5 µA cm^−2^. The consistency of Δ*j* across the 120, 60, and 15-min hybridization times confirms that a 15 min hybridization period is sufficient for detecting miR-21 hybridization events using the ssDNA-21 bioreceptor.

### 3.4. miR-21 Detection with PhP-Au/AuNPs/ssDNA Biosensor

**Figure 6:**
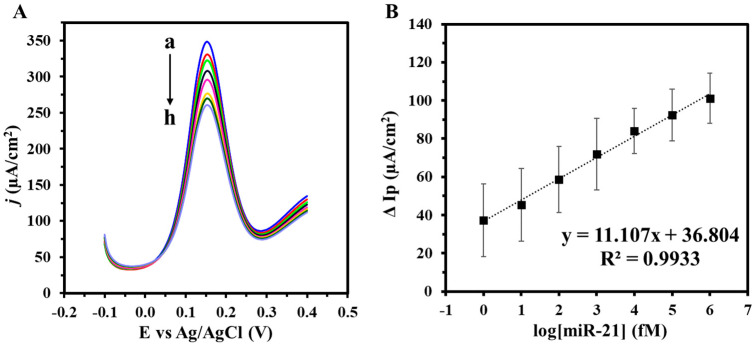
(**A**) SWV of varying target miR-21 concentrations (a: 0 M, b: 1fM, c: 10 fM, d: 100 fM, e: 1 pM, f: 10 pM, g: 100 pM, h: 1 nM) and (**B**) corresponding calibration curve. Voltammograms were obtained in 5 mM (K_3_Fe(CN)_6_^4−/3−^) + 0.1 M KCl. Experiments were conducted in duplicates.

To assess the analytical performance and determine the limit of detection (LOD) of the PhP-Au/AuNPs/ssDNA biosensor, SWV was conducted with miR-21 concentrations ranging from 1 fM to 1 nM utilizing the optimized conditions to validate the CV results. Figure 6A shows the SWV voltammogram depicting current density outputs for the biosensor across the various miR-21 concentrations. The corresponding linearity plot is presented in Figure 6B. Notably, a linear relationship is observed where higher miR-21 concentrations correlate with greater decreases in Ip, underscoring the biosensor’s effective detection capabilities with a sensitivity of 7.69 fM µA^−1^ cm^−2^. The LOD was calculated to be 0.53 fM, significantly lower than many recent gold-based electrochemical biosensors for miR-21 detection (Table 2). The LOD is calculated using 3.3xσ/S, where σ is the standard deviation from the two blank sample measurements, and Sx is the slope of the calibration curve. As shown in Table 2, the fabricated paper-based DNA electrochemical biosensor’s straightforward design, combined with its wide linear range and low LOD, offers a practical solution for scalable production of efficient electrodes for miR-21 biosensing compared to more complex and costly alternatives [42,43,44,45,46,47,48].

### 3.5. Selectivity, Stability, Repeatability, and Reproducibility Testing

**Figure 7:**
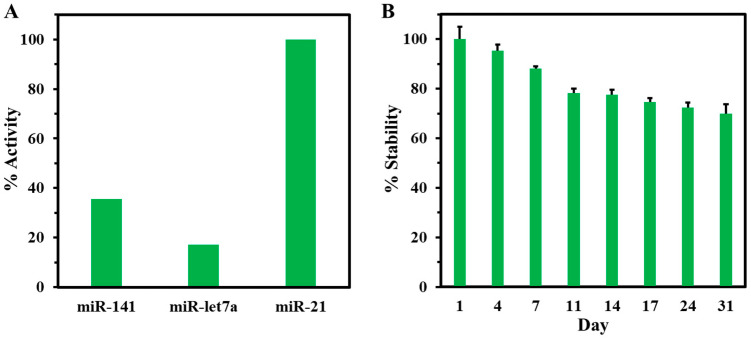
Selectivity and stability of the electrochemical biosensor. (**A**) % Activity of miR-21 biosensor against non-complementary miRs. (**B**) % Stability of PhP-Au/AuNPs/ssDNA vs. days of storage. Experiments were conducted in duplicates.

The selectivity of the fabricated DNA biosensor was conducted in the presence of miR-141 and miR-let7a, common interfering miRs dysregulated in prostate cancer [49]. The three independent PhP-Au/AuNPs/ssDNA electrodes were hybridized with 5 µL of 1 nM miR-let7a, miR-141, or miR-21 for 30 min in a humidity chamber. Figure 7A shows each electrode’s percent activity normalized with miR-21. The decrease in activity observed for miR-141 and miR-let7a was 35.6% and 17.1%, respectively. This significant reduction in activity highlights the biosensor’s strong selectivity toward miR-21.

The stability of the PhP-Au/AuNPs/ssDNA biosensor is examined to showcase the shelf life of the ssDNA-21 capture probe attached to the electrode’s working surface by performing SWV over a period of one month. As shown in Figure 7B, the peak current density decreased by approximately 2% per day over the first 11 days and then plateaued for the remaining 6 days, indicating the ssDNA-21 bioreceptor remained stable by retaining 70% of its original activity for 31 days when stored at 4 °C. The repeatability of the PhP-Au/AuNPs/ssDNA biosensor is examined by performing 15 cycles of CV. The current response change between the first and fifteenth cycles is almost negligible, as shown in Figure S3A. We have tested the reproducibility of three PhP-Au/AuNPs/ssDNA electrodes to compare their current response. As depicted in Figure S3B, the peak current density does not show much variation in the peak current density, with a standard deviation of 13.20 µA cm^−2^.

### 3.6. miR-21 Detection in Serum Samples

**Figure 8:**
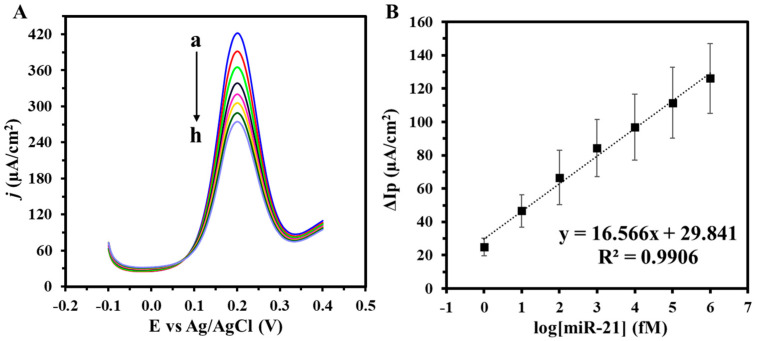
(**A**) SWV of varying target miR-21 concentrations (a: 0 M, b: 1fM, c: 10 fM, d: 100 fM, e: 1 pM, f: 10 pM, g: 100 pM, h: 1 nM) and (**B**) corresponding calibration curve of the target miR-21 concentrations in serum diluted 1000-fold. Voltammograms were obtained in 5 mM (K_3_Fe(CN)_6_^4−/3−^) + 0.1 M KCl. Experiments were conducted in duplicates.

The performance of the DNA biosensor is investigated in serum samples via 1000-fold dilution of miR-21 in 1× PBS over a concentration range from 1 fM to 1 nM. As shown in Figure 8, the biosensor’s response in serum showed similar behavior to miR-21 testing in 1× PBS, wherein the biosensor exhibited a linear electrochemical response over 1 fM to 1 nM miR-21 concentration with a lower LOD of 0.35 fM. The %Recovery and %RSD for the target miR-21 concentrations are shown in Table 3. Therefore, these results showcase the possibility of using our biosensor to analyze a wide range of miR in serum samples.

## 4. Conclusions

We have developed a PhP-Au/AuNPs-based electrochemical biosensor designed for the sensitive detection of miR-21 biomarkers. Our approach involves gold inkjet printing onto photopaper followed by AuNPs electrodeposition, which ensures a high surface area suitable for bioreceptor immobilization and excellent electrical conductivity. The biosensor successfully detects the miR-21 hybridization event with the immobilized complementary ssDNA-21, resulting in a measurable decrease in current density that correlates directly with target miR-21 concentrations. Moreover, the biosensor exhibits good selectivity when tested against other miRs. With a low LOD of 0.35 fM miR-21 in diluted serum, the fabricated biosensor can reliably detect ultralow concentrations of miR-21, which is crucial for early disease diagnosis and monitoring and improving patient health outcomes.

Beyond miR-21, the fabricated gold paper-based DNA biosensor can be adapted for detecting various nucleic acids and proteins, making it a versatile tool in cancer diagnostics. Its simplicity, affordability, rapid response time, and high sensitivity render it particularly suitable for use in resource-limited settings where healthcare infrastructure may be scarce, enabling timely and precise diagnostics to enhance health outcomes and improve global healthcare accessibility.

## Figures and Tables

**Table 1:** Oligonucleotide sequences.

Name	Nucleotide Sequence
ssDNA-21 probe	5′-TCA ACA TCA GTC TGA TAA GCT A/3ThiolMC3
miR-21	5′-UAG CUU AUC AGA CUG AUG
miR-let7a	5′-UGA GGU AGU AGG UUG UAU
miR-141	5′-CAU CUU CCA GUA CAG UGU

**Table 2:** Recent gold-based electrochemical biosensors for detection of miR-21.

No.	Sensor Platform	Technique	Range	LOD	Ref
1	NIPAm-co-AAc microgel/AuNPs	DPV	10 aM–1 pM	1.35 aM	[42]
2	FTO/SWCNTs/den-Au	DPV	0.01 fM–1 µM	0.01 fM	[43]
3	Au	ACV	10 fM–100 nM	3.2 fM	[44]
4	Au/RGO	EIS	1pM–10 nM	300 fM	[45]
5	FTO/APTS/AuPtBNPs	DPV	1 fM–100 nM	0.63 fM	[46]
6	PE/MoS_2_/AuNPs	DPV	135.6–406.8 nM	59.7 nM	[47]
7	3SPCE/GO/GQDs/AuNPs	SWV	1 fM–1 nM	0.04 fM	[48]
8	PhP-Au/AuNPs	SWV	1 fM–1 nM	0.35 fM	This work

NIPAm: N-isopropylacrylamide; AAc: acrylic acid; den-Au: dendritic gold nanocomposite; AuPtBNPs: gold platinum bimetallic nanoparticles APTS: 3-aminopropyltriethoxy silane; MoS_2_: molybdenum disulfide; PE: paper electrode; GQDs: graphene quantum dots.

**Table 3:** %Recovery and %RSD of miR-21in FBS.

MiR-21 Concentration	%Recovery	%RSD
1 fM	67	21
10 fM	103	21
100 fM	113	25
1 pM	117	20
10 pM	115	20
100 pM	121	19
1 nM	125	17

## Data Availability

Data are available upon request.

## Supplementary Materials

The following supporting information can be downloaded at: https://www.mdpi.com/article/10.3390/bios14100485/s1, Figure S1: Construction of PhP-Au/AuNPs electrode; Figure S2: Corresponding linearity plots; Figure S3: Sensor reproducibility.
