# Supplementary material for: Paper-Based DNA Biosensor for Rapid and Selective Detection of miR-21
Source: Biosensors (Basel). 2024 Oct 8;14(10):485. doi: 10.3390/bios14100485 (PMC11506571; doi:10.3390/bios14100485)
Supplement: Supplementary file 1 [file biosensors-14-00485-s001.zip › biosensors-3221965-supplementary.pdf]

Supplementary data

Paper-Based DNA Biosensor for Rapid and Selective Detection of miR-21

Alexander Hunt [1], Sri Ramulu Torati [2] and Gymama Slaughter [1,2,*]

[1] Center for Bioelectronics, Old Dominion University, Norfolk, VA 23508, USA; ahunt038@odu.edu
[2] Department of Electrical and Computer Engineering, Old Dominion University, Norfolk, VA 23508, USA; storati@odu.edu
* Correspondence: gslaught@odu.edu

Inkjet printing parameters: Each electrode of the 60-electrode array has rectangular dimensions of 15 mm × 5 mm and was designed using CoralDRAW. The .bmp file was exported to the DMP-2850 inkjet printer to be converted to a .tf file for printing. Double-sided tape was used to secure the photopaper onto the printer platen. The software was then configured with a substrate thickness (photopaper and double-sided tape) of 67 µm, printing layers of 4 layers, and a jetting speed of 50 Hz for printing the electrode array. 650 µL of gold ink was injected into a cartridge tank, followed by capping the cartridge tank with a DMC-11610 (10 pL drop-size) cartridge head. The platen and ink cartridge temperatures were set at 45 °C and 28 °C, respectively. All 16 jets were used during printing with a resolution of 1016 DPI and a jetting voltage of 25 V.

Fabrication of PhP-Au: Individual gold inkjet-printed photopaper electrodes (PhP-Au) were cut out for fabrication. First, a 3 mm × 3 mm piece of gold foil was applied to the top of the PhP-Au electrode as a contact pad. Then, polyimide (PI) tape was added to the backside of the Au-PhP electrode with PI tape exposed on all sides (**Figure S1A**). Next, another piece of PI tape is applied adhesive side down ~4 mm from the bottom of the PhP-Au electrode (**Figure S1B**). Then, a section of the PI tape is removed to allow terminal access to the gold foil contact pad (**Figure S1C-E**). Next, the PI tape is applied to the edges of the working area (**Figure S1F-H**). Excess PI tape is then removed (**Figure S1I**). The working surface of the PhP-Au is cleaned with sulfuric acid by adding the PhP-Au electrode, the external Ag/AgCl reference, and the external platinum wire counter electrodes to 0.05 M H_2SO_4. Then, cyclic voltammetry (CV) was performed for 10 cycles with a potential range of -0.1 to 1.5 V, and a scan rate of 50 mV/s. Once completed, the electrode was rinsed thoroughly with deionized water to remove residual acid and impurities from the working surface and stored in a desiccator to dry. Lastly, AuNPs are electrodeposited onto the working surface (**Figure 1J**).

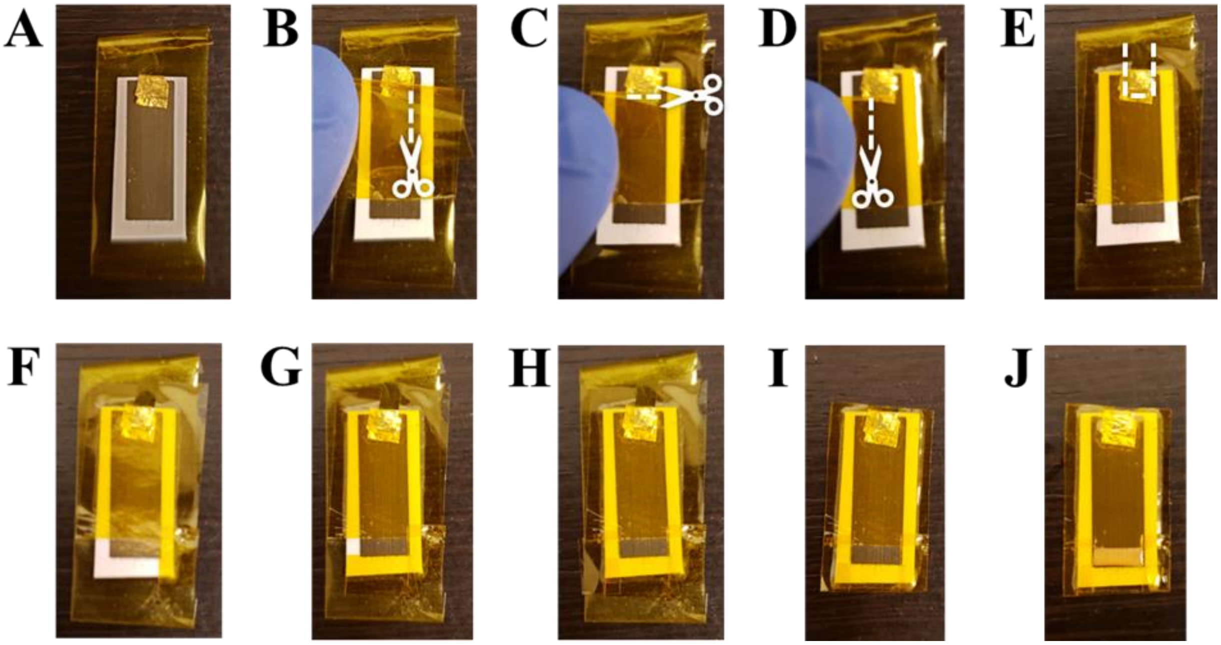

Figure S1. Construction of PhP-Au/AuNPs electrode. (A) the backside of a PhP-Au electrode with a gold foil contact pad adhered to PI tape. (B) PI tape applied adhesive side down ~4 mm from the bottom of the electrode to the bottom of the contact pad. The excess PI tape is folded back and is referred to as the top sections of PI tape. A slit is cut (white dashed line) in the top-right section of the tape and laid to overlap the right side of the contact pad. (C) a slit is cut (white dashed line) where the PI tape meets the bottom of the contact pad. (D) a slit is cut (white dashed line) in the top-left section of the PI tape and laid to overlap the left side of the contact pad allowing terminal access to the contact pad. (E) PI taped excised for terminal access to the contact pad. White dashed lines indicate where the tape was excised. (F) PI tape is applied to the bottom-right corner of the PhP-Au. (G) PI tape is applied to the bottom of the PhP-Au. (H) PI tape is applied to the bottom-left corner of the PhP-Au. (I) excess PI tape is removed from the borders. (J) AuNPs electrodeposited onto the working surface.

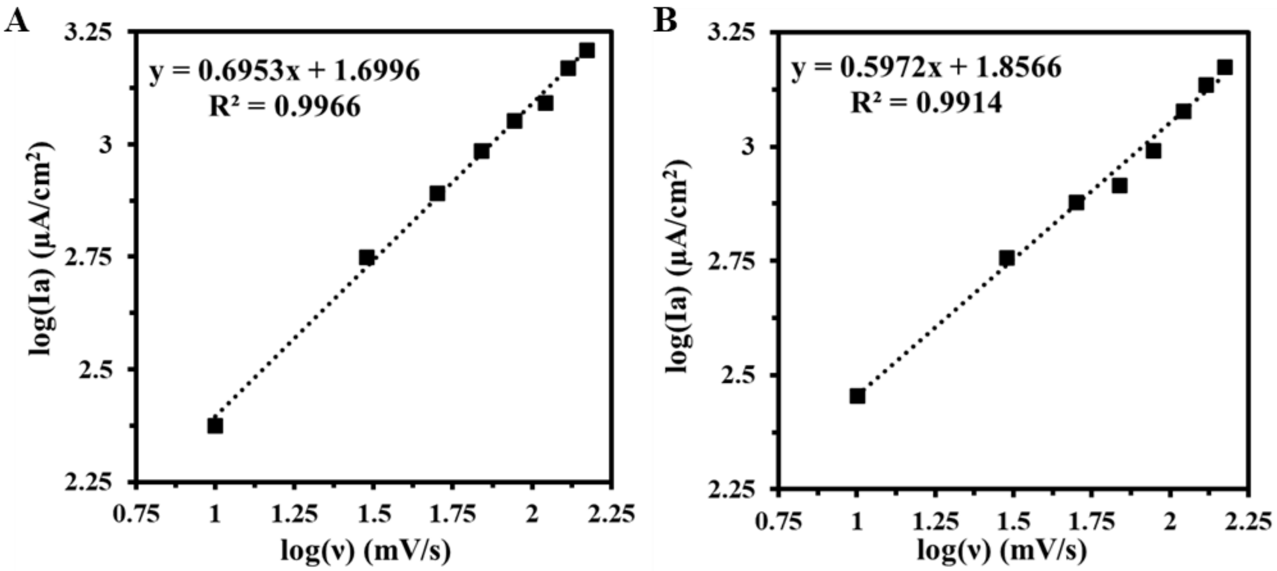

Figure S2. Corresponding linearity plots of (A) PhP-Au/AuNPs and (B) PhP-Au/AuNPs/ssDNA. Voltammograms were obtained in 5 mM ($K_3Fe(CN)_6^{4-/3-}$) + 0.1 M KCl.

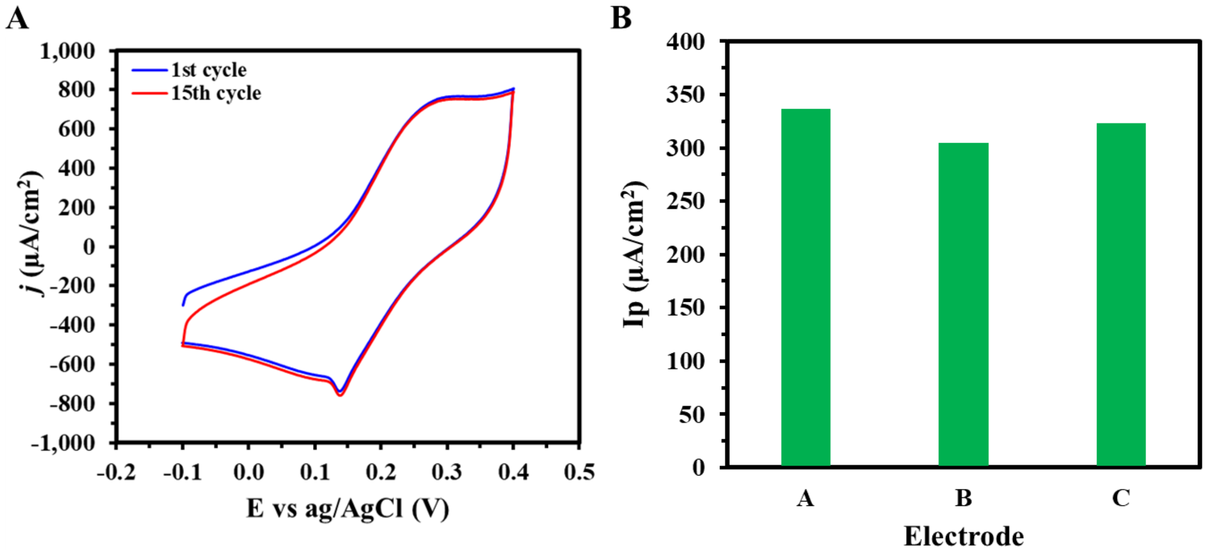

Figure S3. Sensor repeatability and reproducibility. (A) CV overlay of the 1st and 15th cycles performed on PhP-Au/AuNPs/ssDNA electrode. (B) peak current density (I_p) of three independent PhP-Au/AuNPs/ssDNA electrodes. Voltammograms were obtained in 5 mM ($K_3Fe(CN)_6^{4-/3-}$) + 0.1 M KCl.
